# A 21st Century Epidemy-Obesity: And Its Impact on Pregnancy Loss

**DOI:** 10.7759/cureus.12417

**Published:** 2021-01-01

**Authors:** Iana Malasevskaia, Salma Sultana, Aiman Hassan, Azza A Hafez, Fethi Onal, Handenur Ilgun, Stacey E Heindl

**Affiliations:** 1 Obstetrics and Gynecology, California Institute of Behavioral Neurosciences & Psychology, Fairfield, USA; 2 Medicine, California Institute of Behavioral Neurosciences & Psychology, Fairfield, USA; 3 Research, California Institute of Behavioral Neurosciences & Psychology, Fairfield, USA; 4 Anesthesiology and Critical Care, California Institute of Behavioral Neurosciences & Psychology, Fairfield, USA; 5 Anesthesiology, California Institute of Behavioral Neurosciences & Psychology, Fairfield, USA; 6 Neurology, California Institute of Behavioral Neurosciences & Psychology, Fairfield, USA; 7 Medicine, Avalon University School of Medicine, Willemstad, CUW

**Keywords:** obesity, early pregnancy loss, miscarriage, maternal obesity, high body mass index during pregnancy, high body mass index, obesity and pregnancy loss, high bmi, pregnancy surveillance, medical disorders in pregnancy

## Abstract

The prevalence of obesity among women of reproductive age is growing significantly over the last years. Being obese or having a high body mass index (BMI) contributes to many diseases and pregnancy complications. This is concerning as an obese woman is at increased risk for developing several complications during pregnancy and is at increased risk for pregnancy loss, stillbirth, and metabolic disorders of a live-born child in his future. The pregnant woman's body mass index has to be monitored well during the whole pregnancy, and their diet should also be monitored to avoid future complications. Better results can be achieved if every woman would watch their weight before being pregnant for better outcomes in their future pregnancies. This review article aimed to determine the relationship between being obese or having a high BMI and pregnancy loss. Additionally, we tried to find the mechanism that is involved in pregnancy loss in obese women.

## Introduction and background

“We want every pregnancy to have a happy ending” [1]. But unfortunately, it is estimated that as many as 26% of all pregnancies end in miscarriage and up to 10% of clinically recognized pregnancies [2]. Quantifying the full burden of miscarriage is challenging because pregnancy loss rates are higher when pregnancies are clinically recognized [3]. Moreover, spontaneous abortions mainly occur in the first trimester of normal pregnancy [2,3,4]. 

Definition of pregnancy loss 

There are a variety of terms to outline the definition of spontaneous abortion. Therefore, there are some definitions: 

Early pregnancy loss is described by several national organizations as a non-viable intrauterine pregnancy during the 1st trimester (up to 12+6 weeks from the last menstrual period) [5]. Past terminology has included “miscarriage,” “blighted ovum,” and “missed abortion.” [5] 

Early second-trimester pregnancy loss occurs after 13+0 and before 20+0 weeks of gestation [2,4,5,6]. The American College of Obstetricians and Gynecologists (ACOG) estimates that loss of a pregnancy that is less than 20 weeks gestation is the most common form of pregnancy loss [2]. Approximately 20 percent of pregnant women have some bleeding before 20 weeks’ gestation, and one-half will end in spontaneous abortion. Up to 20 percent of pregnancies will end in miscarriage [6]. 

Stillbirth or fetal death is described as pregnancy loss that happens at 20 weeks gestation and later, or baby loss at a weight of 350 grams or greater, is referred to as a stillbirth or fetal death [5,7]. 

The term “abortion” has been recommended to be changed to “spontaneous pregnancy loss” because of acknowledging the emotional aspects of losing a pregnancy [6] 

Risk factors and cause of pregnancy loss 

It is found that almost 50 % of cases of spontaneous pregnancy loss are due to fetal chromosomal abnormalities [2,3,4,6]. In the majority of instances, it is too early to determine the exact cause of pregnancy loss [2]. Several risk factors will increase the risk of pregnancy loss. Advanced maternal age is an important risk factor [2,4,5,6]. Women being at the age of 20 to 30 are at risk of 8.9 % of miscarriage in pregnancy of 20 weeks’ gestation and less, and up to 74.7 percent for women over 40 years old of age [4]. Maternal comorbidities such as antiphospholipid antibody syndrome, thrombophilia, hypertension, diabetes type 1 and 2, celiac disease, thyroid disease, inherited thrombophilia, and systemic lupus erythematosus (SLE) also greatly impact pregnancy loss [2,4,5,7]. Some risk factors can lead to several complications such as pregnancy loss or fetal demise. The risk factors are maternal infections with Parvovirus B19, cytomegalovirus infection (CMV), human immunodeficiency virus (HIV), syphilis, malaria [4,5,7]. Other risk factors that negatively influence pregnancy outcomes are exposure to environmental contaminants such as arsenic, lead, and organic solvents; furthermore, cigarette smoking, large amounts of caffeine use, alcohol and substance use, and some medications [2,4,5,7]. 

Another important and prevalent health concern in obstetrics and gynecology is maternal weight that led to numerous issues during and after pregnancy. Maternal obesity is considered one of the most commonly occurring risk factors seen in obstetric practice [8]. Overweight and obese pregnant women are at a strongly increased risk of miscarriage, whether they conceived after natural conception or assisted reproductive measures [9]. The risk of miscarriage and pregnancy loss before the first liveborn child is 25-37 % higher in obese women [10]. New-born babies of obese mothers are at increased risk of stillbirth, congenital anomalies, prematurity, macrosomia, neonatal death, and are at an increased risk of developing obesity and metabolic disorders in childhood [8,9,10]. In addition to increased risks of pregnancy loss, they are at increased risk for hypertension, preeclampsia, gestational diabetes, thromboembolism, deep vein thrombosis (DVT), postpartum hemorrhage, and reduction in breastfeeding frequency since obesity is associated with reduced prolactin response to suckling [8,10,11]. 

Body mass index (BMI) is established as a person’s weight in kilograms divided by the square of height in meters [12]. The normal BMI, as seen in Table 1, should be around 18.5-24.9. 

**Table 1:** Categories of body mass index BMI - body mass index

BMI	Weight Status
Below 18.5	Underweight
18.5 – 24.9	Normal or Healthy Weight
25.0 – 29.9	Overweight
30.0 and Above	Obese

The Centers for Disease Control and Prevention (CDC) reported in 2016 that the prevalence of obesity in women before pregnancy ranged from 16.6% to 27% [12]. The worldwide prevalence of obesity almost tripled between 1975-2016 [13]. From 1999-2000 through 2017-2018, the prevalence of obesity and severe obesity increased among adults [14]. Almost a quarter of reproductive-age women in the United States are obese with a body mass index of 30 kg/m2 or greater [15]. Obesity is more consistently and strongly associated with pregnancy loss than either diabetes type 1 or type 2 [5]. A meta-analysis of 16 studies done in 2008 demonstrated that a body mass index (BMI) higher than 25 was strongly associated with a nearly 70 % increased odds of spontaneous pregnancy loss after spontaneous or assisted conception [5]. A Danish case-control study of 1644 obese women (BMI ≥30) and 3288 age-matched controls (BMI 19.0-24.9) showed that obese women had a higher incidence of first-trimester miscarriage [odds ratio (OR) 1.2, 95% confidence interval (CI) 1.01-1.46] and recurrent first-trimester miscarriage (OR 3.5, 95% CI 1.03-12.01) [8]. 

Since obesity and overweight among women are increased nowadays, our focus of this review is to evaluate the association between maternal obesity and the risk of pregnancy loss. When considering mechanisms that could influence early pregnancy loss among obese women, it is conceivable that obesity’s effects on the oocyte or embryo could affect the embryo’s potential for development; furthermore, obesity may negatively influence the endometrium, influencing the risk of miscarriage [15]. Since that, we aim to investigate the mechanism and pathophysiology implicated in pregnancy loss in women with a high body mass index. Our goal is to investigate the studies done before showing us the relationship between high body mass and pregnancy loss.

## Review

Obesity: its effect on the embryo, uterus, and ovaries 

Obesity is an inflammatory state in which women have higher circulating levels of C-reactive protein (CRP), which is an inflammatory biomarker [16]. Adipose tissue produces many pro-inflammatory adipokines, including leptin, tumor necrosis factor-alpha (TNFα), and interleukin (IL) 6 [16]. The reproductive tissues are negatively affected by inflammation, like all cells in our body [16]. A possible cause for cellular and organelle damage in obesity is lipotoxicity, a condition where fatty acids from the diet that exceed the adipocytes' storage ability can accumulate in other tissues and cause toxic effects [16]. 

The impact of high body mass on the egg quality and viability 

As we know, ovarian reserve is declining with aging, and the number of oocytes is decreasing. For this reason, any negative environmental exposures may affect the development of the oocyte, which is the capacity of oocytes to be fertilized and support the embryo development [17]. Obesity may affect the woman's fertility by derangement of follicular development, quantitative and qualitative defects of oocyte maturation, impaired fertilization, disrupted meiosis, and mitochondrial dynamics derangements leading to abnormal embryo preimplantation, which is a result of free fatty acids which may be toxic for reproductive tissue [17]. Additionally, obese women have higher levels of circulating free fatty acids, which damage non-adipose cells by increasing reactive oxygen species (ROS), which induce mitochondrial and endoplasmic reticulum (ER) stress resulting in apoptosis of multiple cell types oocytes [17]. While laboratory evidence supports a role for poor oocyte quality as a factor in adverse reproductive outcomes in the setting of obesity, clinical evidence is lacking [11]. 

The impact of high body mass on the embryo 

The preimplantation embryo is also affected in obese women. Comparing human in-vitro fertilization (IVF) cycles with autologous oocytes shows that obese women are more likely to create poor quality embryos as an effect of the lipotoxicity of embryonic cells in a similar fashion as described for the oocyte [17]. This effect is related to the chronic low-grade inflammatory state related to obesity, which is demonstrated by the increased levels of circulating CRP and triglyceride and lactate concentrations in follicular fluid and by the increased expression of pro-inflammatory and oxidative stress-related genes [11,17]. This has been documented clinically in obese women undergoing IVF where embryos derived from oocytes fertilized in vitro are of poorer quality than those derived from normal-weight women [11]. 

The impact of high body mass on the endometrium 

Obese and overweight patients had a significantly increased endometrial expression of haptoglobin and also displayed a significant increase in endometrial expression of transthyretin and beta- globulin, which are inflammatory factors [18]. This may provide evidence for occurring endometrial inflammatory reactions in the endometrial linings of obese women and may contribute to their higher risk of miscarriage [18]. 

Obesity is associated with high serum leptin levels due to leptin produced by the adipose tissue, which acts at the level of the ovary and of the endometrium where it inhibits both human granulosa and thecal cell steroidogenesis, and interferes with the development of the dominant follicle and oocyte maturation, consequently alters endometrial epithelium receptivity [19]. Leptin, behind modulating endometrial receptivity, also exerts a regulatory role in remodeling the endometrial epithelium and stimulating proliferation and apoptotic cell pathways [19]. 

Leukemia inhibitory factor (LIF) is shown to be implicated in implantation regulation, and a significant negative association between endometrial glandular LIF and BMI has been observed [20]. It has also been recommended that a state of relative hyper-estrogenaemia which is seen in women with high BMI (due to aromatization of androgens to estrogen in adipose tissue) may also have a detrimental effect on receptivity [20].

A major pathologic factor could be related to the impaired stromal decidualization in obese women responsible for placental abnormalities, stillbirth, and preeclampsia. However, the most frequent cause of first trimester miscarriages, usually related to embryo chromosomal pathologies, does not increase in overweight women [17]. 

Some researchers have identified a reduction in implantation rates among obese women, whereas others have not demonstrated a weight-related reduction [21]. This suggests that whilst the endometrium may play a part, oocyte quality is likely to be the more influential factor [21]. 

Somatotrophic axis and obesity 

GH (growth hormone) stimulates the growth of small follicles and prevents their atresia, collaborates with gonadotrophins in stimulating later stages of folliculogenesis and luteinization, and facilitates selection and development of dominant follicle [19]. Additionally, GH increases estrogens and progesterone's ovarian production and stimulates endometrium and myometrium in the uterus, all mechanisms that are a prerequisite for successful reproduction [19]. 

Decreased plasma GH levels characterize obese patients due to reduced GH secretion and an increased GH clearance rate [19]. Many factors participate in reducing plasma GH levels, in particular dysregulation of GH-releasing hormone (GHRH), somatostatin (SS), and ghrelin pathways, as well as hyperinsulinemia and excess of circulating free fatty acids (FFA) [19]. 

The effects of obesity and high BMI on pregnancy loss and miscarriage 

Given the recognized impact of obesity on the embryo, the endometrium, and oocytes, it is reasonable to assume that miscarriage rates would be higher in the overweight and obese population [20]. 

Several studies show a relationship between being overweight and a high chance of pregnancy loss and infertility issues. A prospective cohort analysis performed among 18,481 Chinese nulliparous women, using data from a 2006 to 2009 trial by* *Zhou et al. compared with normal weight, obesity was associated with total mortality [adjusted relative risks (ARR) 1.34; 95% CI: 1.03‐1.74] and fetal loss (ARR 1.51; 95% CI: 1.15‐1.99) but not with infant death (ARR 0.53; 95% CI: 0.20‐1.46) [21]. Further analyses showed that obesity was particularly associated with spontaneous abortion (ARR 1.51; 95% CI: 1.13‐2.02) rather than stillbirth (ARR 1.52; 95% CI: 0.65‐3.57) [21]. 

Furthermore, the combined 19,160 cross-sectional studies were utilized by* *Ghimire et al. in Nepal Demographic and Health Survey (NDHS) for the years 2001, 2006, 2011, and 2016 [22]. Miscarriage was defined as a spontaneous pregnancy loss that occurred before the fetus reached seven months of gestational age [22]. The odds of miscarriage were found to be 1.45 times higher than in women with normal-weight adjusted odds ratio (AOR) = 1.45; 95%Cl: 1.06, 1.98, P = 0.021 in obese women [22]. 

Additionally*, *Hahn et al. examined the relationship between selected anthropometric factors and risk of spontaneous abortion (SAB) among 5132 women enrolled in a Danish internet-based prospective cohort study of pregnancy planners [23]. After adjustment for potential confounders, the HRs (hazard ratio) for SAB among underweight women (body mass index (BMI), kg/m (2)) <20), overweight (BMI: 25-29) and obese (BMI ≥30) women were 1.00 [95% CI: 0.81, 1.24], 0.90 [95% CI: 0.73, 1.09] and 1.23 [95% CI: 0.98, 1.54], respectively, compared with normal-weight women (BMI 20-24) [23]. The association among obesity and SAB was stronger for early SAB (<8 weeks gestation); HR: 1.34 95% CI: 1.01, 1.77 [23]. 

Another prospective cohort study by Al-Hakmani et al., which was conducted among pregnant Omani women in antenatal clinics in their first trimester in the Seeb province [24]. Seven hundred pregnant women were enrolled and were grouped according to their BMI: 245 (35%) were normal weight, 217 (31%) were overweight, and 238 (34%) were obese [24]. Miscarriages were more common in obese women, 11.9% (n = 27) compared to the women with normal weight and (6.7% and 9.4%, in overweight groups, respectively) [24]. 

A cross-sectional study that used data of Brazilian women aged between 15-45 years old, data from the National Demographic and Health Survey in 2006 by Felisbino-Mendes et al. was done [25]. In that study body mass index (BMI), waist circumference (WC), and waist-to-height ratio (WHR) [25] were measured. The survey data was used to assess the relationship between obesity and the study outcomes [25]. Three obesity markers were found to be strongly and positively associated with spontaneous abortion and stillbirth occurrence; there was a strong affirmation that for every unit increase in BMI (OR = 1.05; 95%CI: 1.02-1.08) and WHR (OR = 1.32; 95%CI: 1.03-1.69), the odds to have a spontaneous abortion was higher [25]. 

Lately, to compare the incidence of spontaneous miscarriage in women with moderate to severe obesity to that in women with a normal BMI, a prospective cohort study was done by O'Dwyer et al. by sonographic confirmation of the fetal heart rate in the first trimester [26]. Of the 3,000 women enrolled, the miscarriage rate overall was 3.9% (n = 117), the mean gestational age at enrolment was 11.1 weeks [26]. In class two and three (BMI > 34.9 kg/m2 obese primigravida the miscarriage rate was 11.3% (n = 8) compared with 2.7% (n = 24) in the normal BMI category (p = 0.003), and 3.7% (n = 5) in the class one obese category (not significant), as shown in Table 2 [26]. 

 

**Table 2:** Review of the studies and results. The relationship between obesity and high body mass index and pregnancy loss. BMI: body mass index; CI: confidence interval

Zhou et al. [21]	A prospective cohort analysis	data from a 2006 to 2009	18,481 Chinese nulliparous women	Obesity was defined as BMI ≥ 27.5 kg/m2	Results: fetal loss (ARR 1.51; 95% CI: 1.15-1.99 spontaneous abortion (ARR 1.51; 95% CI: 1.13-2.02)
Ghimire et al. [22]	The combined 19,160 cross-sectional pregnancy data from the Nepal Demographic and Health Survey (NDHS)	Years 2001, 2006, 2011 and 2016 were utilized	19,160 cross-sectional pregnancy data		The odds of miscarriage were 1.45 times higher (Adjusted odds ratio (AOR) = 1.45; 95%Cl: 1.06, 1.98, P = 0.021) among women with obesity
Hahn et al. [23]	Danish Internet-based prospective cohort study	2014	5132 women	Overweight (BMI: 25-29) and obese (BMI ≥30) Cox proportional hazards regression models, was used with weeks of gestation at the time scale, to compute hazard ratios (HRs) of SAB and 95% confidence intervals (CIs)	After adapting for potential confounders, the HRs for SAB among underweight (BMI, kg/m (2)) <20), overweight (BMI: 25-29), and obese (BMI ≥30) women were 1.00 [95% CI: 0.81, 1.24], 0.90 [95% CI: 0.73, 1.09] and 1.23 [95% CI: 0.98, 1.54], respectively, compared with normal-weight women (BMI 20-24). The relationship between obesity and SAB was stronger for early SAB (<8 weeks gestation); HR: 1.34 95% CI: 1.01, 1.77. The HR for height ≥174 cm vs. <166 cm was 0.81 [95% CI: 0.66, 1.00]. Increased waist-to-hip ratio (WHR) was inversely associated with risk of SAB (HR: 0.81; 95% CI: 0.63, 1.05)
Al-Hakmani et al. [24]	A prospective cohort study	2016	700 pregnant women, Oman	Where 245 (35%) were normal weight, 217 (31%) were overweight, and 238 (34%) were obese	Miscarriages were more common in obese women 11.9% (n = 27) compared to the normal weight and overweight groups (6.7% and 9.4%, respectively)
Felisbino-Mendes et al. [25]	Cross-sectional study using secondary data of Brazilian women of reproductive age (15-45 years old) from the National Demographic and Health Survey	2006		Obesity was measured by (BMI), waist circumference (WC) and waist-to-height ratio (WHR)	The three obesity markers used were positively and strongly associated with stillbirth and spontaneous abortion occurrence. There was strong proof that for each unit of increased BMI (OR = 1.05; 95%CI: 1.02-1.08) and WHR (OR = 1.32; 95%CI: 1.03-1.69), the odds of having a spontaneous abortion was higher
O'Dwyer et al. [26]	A prospective observational study	2012	3,000 women enrolled,	The mean gestational age at enrolment was 11.1 weeks by ultrasound.	In the class 2-3 (BMI > 34.9 kg/m (2)) obese primigravida the miscarriage rate was 11.3% (n = 8) compared with 2.7% (n = 24) in the normal BMI category (p = 0.003), and 3.7% (n = 5) in the class 1 obese category (not significant).

## Conclusions

Based on the above review study, there is a clear relationship between obesity and pregnancy loss, revealed from several studies. It is shown that there are strong negative effects of high BMI on pregnancy outcome and a high percentage of pregnancy loss. There is a clear negative effect of obesity on the quality of women's oocytes, embryos, and hormones with high body mass. Poor endometrial receptivity is also one of the theories that some researchers argue about, but there is no strong evidence and studies that could strongly confirm this. Despite several studies that were done on obesity and pregnancy loss, there still a gap in research. There is a demand for further research on the endometrial and placental cause of pregnancy loss in obese women. Several studies estimate that cause of pregnancy loss can be poor endometrial receptivity, high inflammatory state as a result of cytokines, hormonal imbalance, or poor blood supply of the endometrium and placenta; however, still no definitive answer for this question. Future research is needed to verify these theories.

Consequently, we believe that obese women should be better monitored and encouraged to control their BMI and diet. Changing their lifestyle should also be advised beyond pregnancy. If there is a need for a specialist or a dietician consultation, they should be provided with, and an exercise plan should also be arranged. Despite an enormous improvement in science and endocrinology, there are still numerous obese pregnant women struggling with high BMI and pregnancy loss. Further studies should be conducted on endocrinology and physiopathology of obese pregnant women, which will help women manage and treat them accordingly. Additionally, further studies on weight loss and pregnancy outcomes would also be beneficial. 
